# Differential Time Course of LVEF Recovery Between Conduction System Pacing Versus Biventricular Pacing

**DOI:** 10.1016/j.jacasi.2026.03.020

**Published:** 2026-05-06

**Authors:** Hiroyuki Kato, Satoshi Yanagisawa, Taku Sakurai, Kazumasa Suga, Hisashi Murakami, Kenji Kada, Naoya Tsuboi, Ryusuke Ota, Yasuya Inden, Toyoaki Murohara

**Affiliations:** a Department of Cardiology, Japan Community Healthcare Organization Chukyo Hospital, Nagoya, Japan; b Department of Cardiology, Nagoya University Graduate School of Medicine, Nagoya, Japan

**Keywords:** cardiac resynchronization therapy, His-bundle pacing, left bundle branch area pacing, responder, reverse remodeling

Conduction system pacing (CSP), comprising His-bundle pacing (HBP) and left bundle branch area pacing (LBBAP), is a promising alternative to biventricular pacing (BVP) for cardiac resynchronization therapy (CRT). Previous studies have shown greater improvement in left ventricular ejection fraction (LVEF) with CSP than BVP, based on echocardiographic assessments during short-term follow-up (6-12 months).^1^, ^2^, ^3^, ^4^ However, whether there are differences in LVEF and clinical outcomes between the 2 pacing strategies over longer-term follow-up remains unclear. We compared long-term LVEF improvements and clinical outcomes between CSP and BVP in patients with nonischemic cardiomyopathy (NICM) associated with left bundle branch block (LBBB).

We examined 30 consecutive patients with sinus rhythm and NICM associated with LBBB fulfilling the Strauss criteria who underwent CRT using either CSP or BVP at Chukyo Hospital between February 2017 and February 2024 (Figure 1A). Patients with a Class I indication for an implantable cardioverter-defibrillator (ICD) or those selected to receive an ICD device were excluded.^5^ The study was approved by our Institutional Ethics Committee and was conducted in accordance with the Declaration of Helsinki principles. Written informed consent was obtained from all patients. CSP was performed using standard implantation techniques.^1^^,^^6^ Success criteria were His-bundle capture with LBBB correction threshold ≤1.5 V for HBP and left bundle branch capture with threshold ≤2.0 V for LBBAP.^6^ Left bundle branch capture was confirmed by an output-dependent QRS transition from nonselective to selective or myocardial only capture. All patients received dual-chamber or CRT pacemaker devices. Patients in the CSP group were assigned to CSP only. The atrioventricular interval for LBBAP was programmed to allow fusion with intrinsic right bundle branch conduction. In the BVP group, fusion optimization algorithms were permitted. Fusion-optimized intervals were adjusted as needed to achieve the narrowest QRS duration. Echocardiography was performed at the 6-month, 1-year, 2-year, and 3-year follow-up periods. Clinical outcomes included all-cause mortality and heart failure hospitalization (HFH). HFH was defined as an unplanned emergency department visit or inpatient hospitalization for worsening heart failure that required intravenous diuretic therapy. Statistical significance was set at *P <* 0.05. Analyses were performed using SPSS (version 20, IBM) and R (version 4.4.2).

**Figure 1:**
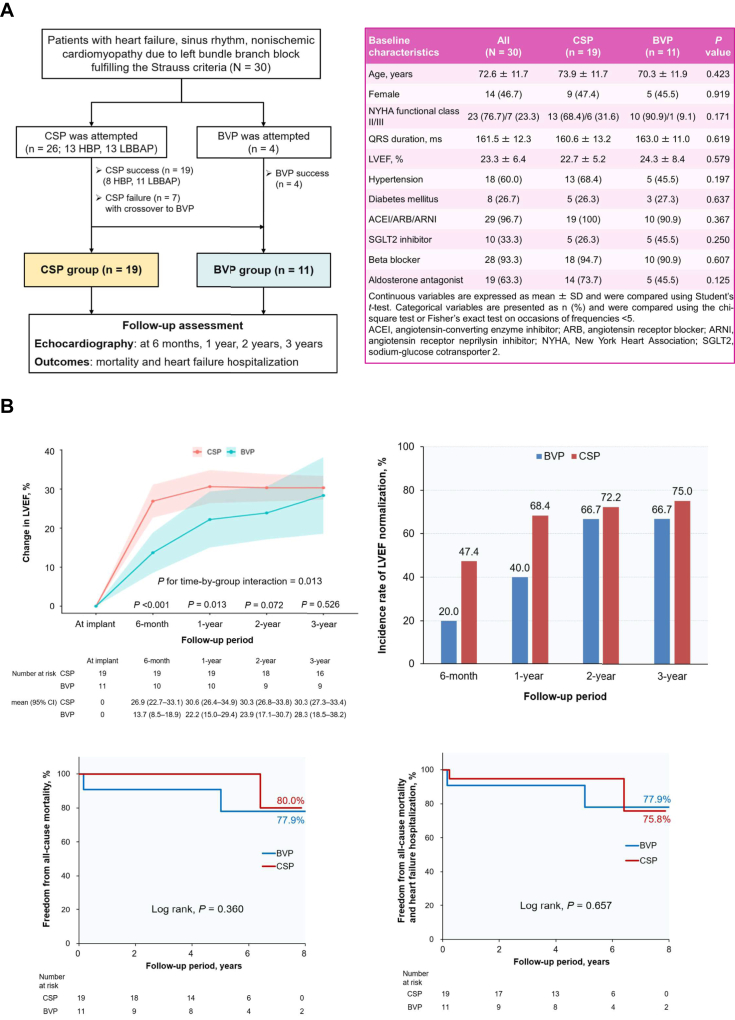
Study Population and Outcomes (A) Study flowchart and baseline characteristics. (B) Longitudinal left ventricular ejection fraction (LVEF) changes (solid lines represent mean values and shaded areas indicate 95% CIs), incidence of LVEF normalization, and Kaplan-Meier survival curves for all-cause mortality and composite endpoint in patients treated with conduction system pacing (CSP) vs biventricular pacing (BVP). *P* values indicate comparisons between the 2 groups. The time-by-group interaction of longitudinal LVEF changes was evaluated using a linear mixed-effects model with random intercepts for subjects. Degrees of freedom were estimated using Satterthwaite’s method. The time-by-group interaction was statistically significant in an unadjusted model *(P =* 0.013) and a model adjusted for age, sex, and baseline QRS duration *(P =* 0.012). The clinical outcomes are presented as Kaplan-Meier curves, and significance was assessed using log-rank tests. ACEI = angiotensin-converting enzyme inhibitor; ARB = angiotensin receptor blocker; ARNI = angiotensin receptor neprilysin inhibitor.

CSP was attempted in 26 of 30 patients and was successful in 19 (8 HBP and 11 LBBAP). Selective conduction system capture was intraoperatively documented in 6 of 8 (75.0%) HBP and 6 of 11 (54.5%) LBBAP procedures. BVP was achieved in all 11 patients, including 7 with unsuccessful CSP attempts. No procedure-related complications were observed in either group. Baseline clinical characteristics did not differ significantly between the 2 groups (Figure 1A). The paced QRS duration was significantly shorter in the CSP group than in the BVP group (115.5 ± 12.6 ms vs 138.6 ± 12.6 ms; *P <* 0.001). No complications requiring discontinuation of pacing therapy or lead revision occurred in either group.

All patients had ≥5% LVEF improvement and ≥15% reduction in left ventricular end-systolic volume at 6 months, indicating a favorable response to CRT. There was a significant difference in the 3-year time course of LVEF improvement between the 2 groups: LVEF improved more rapidly within 1 year in the CSP group, whereas the BVP group showed slower but continuous improvement throughout the 3 years *(P =* 0.013) (Figure 1B). At 1 year, LVEF normalization (defined as an LVEF of ≥50%) was observed in 13 of 19 (68.4%) vs 4 of 10 (40.0%) in the CSP and BVP groups, respectively. However, the corresponding rates at 3 years were 12 of 16 (75.0%) and 6 of 9 (66.7%), with the intergroup difference diminishing over time (Figure 1B). During a median follow-up of 5.3 years (Q1-Q3: 3.6-7.1 years), 3 deaths (CSP, n = 1; BVP, n = 2), and 1 HFH (CSP, n = 1) occurred. Freedom from all-cause mortality was comparable between the groups (Figure 1B). The 5-year freedom from the composite endpoint did not differ significantly between the CSP and BVP groups (94.7% vs 90.9%; log-rank *P =* 0.298) (Figure 1B). No malignant ventricular arrhythmias were observed.

Previous studies have shown that CSP yields earlier and greater CRT effects than BVP; however, most had short follow-up periods and broad inclusion criteria, encompassing patients with atrioventricular block, ischemic cardiomyopathy, intraventricular conduction disturbances other than LBBB, and those requiring an ICD.^1^, ^2^, ^3^, ^4^^,^^7^, ^8^, ^9^ In this study, CSP was associated with greater LVEF improvement at 6 months compared with BVP (26.9% vs 13.7%), consistent with the findings of the LBBP-RESYNC randomized study that compared the CRT effects of LBBAP vs BVP in patients with NICM and LBBB (23.3% vs 15.9%).^3^ However, this difference was almost gone at the 3-year follow-up (30.3% vs 28.3%). In contrast to LVEF improvement with BVP, which exhibits gradual reverse remodeling over 3 to 5 years,^10^ CSP demonstrated rapid electrical resynchronization and LVEF normalization at approximately 1 year. Despite the differential time courses of LVEF recovery, the long-term clinical outcomes were comparable between the 2 groups, consistent with some previous studies.^2^^,^^4^ This may be because CRT was highly effective in our population, which was characterized by sinus rhythm, NICM, LBBB, and no Class Ⅰ indication for ICD therapy. Unexpectedly, no difference in 1-year freedom from HFH (log-rank *P =* 0.468) was found, contrasting that in a previous study,^8^ warranting further investigation into the prognostic impact of CSP-related rapid electrical resynchronization.

This study adds to the evidence for CRT strategies in Asian populations by offering long-term follow-up data (median 5.3 years), highlighting temporal variations in reverse remodeling and demonstrating that CRT continues to be highly effective, as exemplified by the high rate of LVEF normalization. This study has some limitations, including its small sample size and nonrandomized design. Of the 11 patients assigned to the BVP group, 7 were crossover cases caused by unsuccessful CSP, introducing a potential selection bias. Larger randomized studies are required to validate the current findings. Whether CSP is superior to BVP in CRT-eligible patients remains a key clinical question. Longer comparative follow-up periods and strict patient stratification may be essential for addressing this issue.

## Funding Support and Author Disclosures

The authors have reported that they have no relationships relevant to the contents of this paper to disclose.
